# Lifestyle modification and metformin as long-term treatment options for obese adolescents: study protocol

**DOI:** 10.1186/1471-2458-9-434

**Published:** 2009-11-30

**Authors:** A Justine Wilson, Harry Prapavessis, Mary E Jung, Anita G Cramp, Joy Vascotto, Larissa Lenhardt, J Kevin Shoemaker, Margaret Watson, Tracy Robinson, Cheril L Clarson

**Affiliations:** 1 Department of Health Sciences, School of Kinesiology, The University of Western Ontario, London, Ontario, Canada; 2 Faculty of Medicine, The University of Western Ontario, London, Ontario, Canada; 3 Children's Hospital, London Health Sciences Centre, Ontario, Canada; 4 Healthy Eating and Activity Program, Children's Hospital, London Health Sciences Centre, Ontario, Canada

## Abstract

**Background:**

Childhood obesity is a serious health concern affecting over 155 million children in developed countries worldwide. Childhood obesity is associated with significantly increased risk for development of type 2 diabetes, cardiovascular disease and psychosocial functioning problems (i.e., depression and decreased quality of life). The two major strategies for management of obesity and associated metabolic abnormalities are lifestyle modification and pharmacologic therapy. This paper will provide the background rationale and methods of the REACH childhood obesity treatment program.

**Methods/design:**

The REACH study is a 2-year multidisciplinary, family-based, childhood obesity treatment program. Seventy-two obese adolescents (aged 10-16 years) and their parents are being recruited to participate in this randomized placebo controlled trial. Participants are randomized to receive either metformin or placebo, and are then randomized to a moderate or a vigorous intensity supervised exercise program for the first 12-weeks. After the 12-week exercise program, participants engage in weekly exercise sessions with an exercise facilitator at a local community center. Participants engage in treatment sessions with a dietitian and social worker monthly for the first year, and then every three months for the second year. The primary outcome measure is change in body mass index and the secondary outcome measures are changes in body composition, risk factors for type 2 diabetes and cardiovascular disease, changes in diet, physical activity, and psychosocial well-being (e.g., quality of life). It is hypothesized that participants who take metformin and engage in vigorous intensity exercise will show the greatest improvements in body mass index. In addition, it is hypothesized that participants who adhere to the REACH program will show improvements in body composition, physical activity, diet, psychosocial functioning and risk factor profiles for type 2 diabetes and cardiovascular disease. These improvements are expected to be maintained over the 2-year program.

**Discussion:**

The findings from this study will advance the knowledge regarding the long-term efficacy and sustainability of interventions for childhood obesity.

**Trial Registration:**

ClinicalTrials.gov number NCT00934570

## Background

Childhood obesity is one of the most prevalent health issues affecting children in developed countries globally. The International Obesity Task Force [1] reported that there are more than 155 million children worldwide who are overweight or obese. Over the past 3 decades, the rate of adolescent obesity has tripled in Canada, from 3% in 1978 to 9% in 2004 [2]. The significance of this emerging trend is the connection between childhood obesity and type 2 diabetes (T2D), cardiovascular disease (CVD), psychosocial problems (i.e., depression and low self-esteem) and adult obesity [3-5]. A reduction in obesity and associated risk factors may prevent T2D and CVD in this high-risk pediatric population [4,6]. Two major strategies for management of obesity and associated metabolic abnormalities are lifestyle modification [7] and pharmacologic therapy [6].

Wilfley and colleagues [7] conducted a meta-analytic review of RCTs for the treatment of childhood obesity and concluded that lifestyle interventions were effective at decreasing participants' body mass indexes (BMI) in the short-term and reported some evidence for long-term positive effects. Interestingly, Wilfley and colleagues [7] found that wait-list or control group participants increased in percentage overweight from 2.1% (immediately following treatment) to 2.7% (at follow-up). Thus, without treatment, it can be expected that the average participant will continue to gain weight. These findings also suggest that treatment programs that result in slight improvements or maintenance of participant's percentage overweight should be utilized.

Although positive findings were reported for improvements in percentage overweight, Wilfley and colleagues point out a number of limitations to the RCTs reviewed and make several recommendations for future research. Their first recommendation is that treatment programs implement sufficient long-term follow-up assessments, occurring at least 1-year and ideally 2-years post-randomization. Longer-term follow-ups allow for the determination of factors that protect and/or are associated with diminished treatment effects over time. Wilfley and colleagues [7] also suggest that in order to accurately assess changes in participants' percentage overweight, childhood obesity treatment studies should utilize weight outcome assessments that take into account changes in height. Reporting results that highlight the clinical importance of the findings, including comprehensive participant characteristics, analyses used, and other measures of health and psychosocial functioning (i.e., effects on psychological factors and comorbidities, such as T2D) is also recommended [7]. Finally, they suggest that future studies elucidate variables that moderate and/or mediate the long-term effectiveness of childhood obesity treatment programs [i.e., environmental or psychological factors; [7]].

### Importance of Theory-based Interventions

In developing an intervention aimed at changing individuals' behaviour, it is important to use behaviour change theories and models to guide the intervention. Based on established principles, theory-based interventions aid in the planning, implementing and evaluating stages of an intervention [8]. Furthermore, theory-based interventions allow researchers to make predictions about why certain changes occur and help determine potential mechanisms through which the intervention is working [8]. For example, theories of behaviour change suggest that psychosocial factors play an important role in changing behaviour. In addition, theories of behaviour change suggest important variables to measure and methods of assessing the intervention. In doing so, researchers are able to identify specific aspects of the intervention that worked, and aspects that need improvement.

One of the most influential theories of health behaviour change is social cognitive theory [SCT; [9]]. SCT describes human behaviour as an interaction between the individual, behaviour and environment and has been successfully used to understand, predict and change behaviour [10]. Therefore, an SCT-based childhood obesity intervention would focus on individuals and their behaviour, as well as the environment (i.e., the individual's family and their living situation) in order to help them make healthy lifestyle changes. A model that incorporates these variables is the family-based multidisciplinary obesity treatment model [11]. This model was developed over 25 years ago and has demonstrated both short- and long-term efficacy [12]. Families are generally referred to these programs based on one (or more) of the children's health status (i.e., high blood lipid levels or high BMI). Typically, multidisciplinary family-based treatment programs involve a psychologist or social worker, a pediatrician, a dietitian and a physical trainer. In line with SCT, the health care providers teach the families ways to change their behaviours for their particular living situation. For example, behaviour modification techniques taught in such programs incorporate self-regulation skills, including goal setting, self-monitoring, and corrective behaviours (e.g., including three of four food groups in each meal, planning for regular physical activity).

Multidisciplinary family-based childhood obesity treatment programs are able to address a number of the limitations outlined in the Wilfley and colleagues review. For example, such treatment programs are structured to involve long-term treatment and follow-up. As previously mentioned, these programs involve specialized health care providers (i.e., behavioural, physical, nutritional, psychological and medical), which allows for more thorough assessments to be administered (i.e., markers of T2D and CVD, detailed dietary reviews, objective assessments of physical activity). Furthermore, the interpretation and application of results can be more detailed and clinical significance can be disseminated because all healthcare providers are experts in their field. The specific components of multidisciplinary childhood obesity treatment programs will be discussed.

### Behaviour Change Skills Sessions

One of the main goals of a childhood obesity treatment program is to help participants lose weight. Research has demonstrated that obesity interventions have been able to achieve weight loss in the short term [7,12,13], and there is some evidence to suggest they achieve long-term weight loss as well [12]. In order for participants to sustain their weight loss, it is essential for obesity treatment programs to teach participants skills to maintain their behaviour changes. The underlying skill in making and maintaining such lifestyle changes is the ability to self-regulate health behaviours, which is the ability to consciously make healthy choices [i.e., engage in regular physical activity and healthy eating; [14]]. According to SCT theory, self-regulatory skills play an important role in making and maintaining behavioural changes [14,15]. Self-regulation involves monitoring one's behaviour, comparing one's behaviour to set goals or criteria (evaluation/feedback) and changing the behaviour to meet the set goals [15]. A recent study by Alm and colleagues [16] found that overweight adolescents who weighed themselves regularly (self-monitoring) engaged in more healthy weight control behaviours (self-regulation), including decreasing their caloric intake, and eating less fatty foods and junk foods. However, effectively teaching these skills can be difficult. Recently, Brawley, Rejeski and Lutes [17] developed the group-mediated cognitive-behavioural (GMCB) model, which aims to facilitate learning of self-regulation skills through using the group as an agent of change. The GMCB model involves teaching self-regulatory skills throughout the sessions, and increasingly relies on independent self-regulation to teach participants to be independently active.

GMCB interventions are based on SCT [9] and the group dynamics literature [18]. SCT is used as the theoretical framework because it suggests that to increase adherence to an optional behaviour it is essential to change an individual's cognitions. For example, if the individual is to change their behaviour they must value the outcome of the behaviour, believe they can produce the desired outcome, and believe that the outcome will result from successfully completing the behaviour [19]. Group dynamics theory is used because group cohesion - a central component of group dynamics, is associated with greater adherence to physical activity [20]. Thus, developing a cohesive group is thought to be one of the underlying mechanisms to motivate and enhance learning of behaviour change skills (i.e., self-regulation and goal setting).

Recently, separate studies involving post-natal women [21] and elderly adults [17,22,23] implemented GMCB interventions, and found a positive impact on adherence to an exercise program. These GMCB interventions lead to greater improvements in frequency of exercise [21-23], long-term adherence [21-23], fitness [22], self-efficacy for mobility [22], and barrier self-efficacy [21], as compared to those in control groups. Collectively, GMCB studies are thought to be effective because they facilitate learning self-regulatory skills through a cohesive and supportive group environment, which encourages participants to make and maintain healthy lifestyle changes.

### Physical Activity

One of the most frequently cited factors contributing to the current childhood obesity epidemic is the high rate of physical inactivity in youth today [24,25]. The Public Health Agency of Canada [26] suggests that youth should accumulate at least 90 minutes of moderate and vigorous physical activity per day, and decrease the amount of time spent being sedentary (i.e., watching TV) by at least 90 minutes per day. Disappointingly, based on more conservative guidelines of only 30 minutes of physical activity per day, Sithole and Veugelers [26] found that only 48.7% of Canadian children are active at least at a moderate intensity. Thus, one of the ways to address the childhood obesity epidemic is to get obese children more physically active. Multidisciplinary obesity treatment programs often involve a specific exercise component, ranging from telling people to start exercising, to having people exercise while at the program [11,12]. van Sulijs and colleague's [27] review found that more intensive physical activity interventions were associated with greater improvements in physical activity levels. Further, it is suggested that programs that involve structured physical activity sessions are more likely to be more intensive. Thus, including physical activity sessions in the program may aid in the success of the treatment program.

### Nutrition

Nutrition also plays an important role in the development of childhood obesity. Indeed, consuming a diet rich in foods that are lower in nutrient density and fiber and higher in fat and calories, contributes to excessive weight gain [28]. According to the Ontario Ministry of Health and Long Term Care [29], only 42% of individuals age 12 years and older reported consuming the recommended five or more servings of fruits and vegetables per day. Diets that include a variety of and considerable amount of fruit and vegetable servings are associated with healthy weights, a decreased risk of obesity as well as a decreased risk of other diseases such as cancer and cardiovascular disease [30-32]. Accordingly, nutrition counseling that aims to improve eating habits (i.e. increase fruit and vegetable servings, limit lower nutrient density and higher fat and calorie foods) is a critical component of a successful obesity treatment program [28].

### Family Coaching

Given the necessary influence of parents throughout the development of their children, it is essential to involve parents in the treatment of childhood obesity [11]. Based on Bandura's SCT and Epstein's model of family-based interventions, parental modeling (i.e., parents engaging in regular healthy eating and exercise) and parental reinforcement (i.e., parents supporting children in their healthy changes), play an essential role in their child's weight loss. Thus, multidisciplinary treatment programs often involve a family coach (i.e., a social worker or psychologist), who works with the family to address family issues and help parents set a good example for their children in making healthy changes.

In addition to involving the parents, the family coach addresses important issues the child is facing. Childhood obesity research often explores issues of diminished self-esteem and body image, and how they relate to increased weight. Research suggests a strong association between lowered self-esteem and higher BMI measures for elementary school-aged children [33]. Furthermore, research suggests that diminished self-esteem is associated with significant increases in sadness, loneliness and nervousness [34]. These issues are addressed during social work sessions, as early adolescence is a critical period for the development of self-esteem among obese children [34].

### Medication

For obese children and adolescents, lifestyle changes alone may have a positive impact. However, the sustainability of these lifestyle changes remains to be evaluated. Preliminary data indicate that the addition of metformin to a lifestyle intervention program is associated with greater reduction in BMI as well as improved modification of metabolic risk factors for T2D and CVD [35]. Metformin often promotes weight loss and is clinically effective in reducing insulin resistance as well as positively impacting lipid levels. The efficacy and safety of metformin in improving BMI and associated metabolic risk factors in obese, non-diabetic adolescents has been demonstrated in several pediatric trials, including a recently published 6 month intervention by Clarson and colleagues [35], in doses up to 2000 mg daily with duration of therapy from 8-26 weeks [36-38]. Clarson and colleagues [35] found that BMI and dyslipidemic profiles improved most in participants taking metformin. However, independent of metformin, lifestyle intervention resulted in improvement in participants' metabolic risk factors (i.e., plasma lipids and adipocytokines).

Although these studies all demonstrated improvement in anthropometry in response to up to 6-months of metformin therapy, none were maintained for longer than 6 months. Data on the long-term efficacy of metformin intervention programs for pediatric obesity are lacking, in particular relating to the sustainability of lifestyle interventions.

### REACH

REACH is a 2-year program including a metformin/placebo intervention, an intensive 12-week exercise program followed by a weekly 1.75 year long-term exercise program, behaviour change techniques, family sessions with a dietitian and a social worker, and comprehensive medical monitoring. The primary outcome measure is change in BMI. The secondary outcome measures are changes in body composition, risk factors for T2D and CVD, diet, PA and psychosocial functioning. It is hypothesized that participants who take metformin and engage in vigorous intensity exercise will show the greatest improvements in body mass index. In addition, it is hypothesized that participants who adhere to the REACH program will show improvements in body composition, physical activity, diet, psychosocial functioning and risk factor profiles for type 2 diabetes and cardiovascular disease. These improvements are expected to be maintained over the 2-year program.

## Methods/design

### Design

The REACH study is a four-arm parallel, randomized placebo-controlled registered trial (ClinicalTrials.gov number NCT00934570). This is a 2-year intervention study on the effects of a structured lifestyle intervention and metformin (or placebo) on BMI and other risk factors for T2D and CVD in obese adolescents (age 10-16 years). Participants are randomized to metformin medication or placebo, and then randomized to engage in a moderate or vigorous intensity exercise program for the first 12 weeks of the 2-year program. Randomization is completed via a computer-generated randomized numbers table. The REACH study coordinator is responsible for enrolling participants and group assignment. All other study personnel are blinded to medication and exercise group assignment, with the exception of the exercise specialist who is aware of exercise group assignment. Assessments are conducted pre- and post-intervention, at 13-weeks, 6-months, 1- and 2-years. This study has been approved by the University of Western Ontario Research Ethics Board (REB # 15590) and Health Canada and informedJW consent is obtained from all study participants and their parent/guardian.

### Participants

Participants are recruited via local pediatricians' referrals, newspaper and radio ads and posters in public centers. Participants are eligible to participate if they: are 10-16 years of age, live within 30 km around London, Ontario, are obese (BMI greater than the 95^th ^percentile for their age and gender) and have no contraindications to exercise or to taking metformin medication. Exclusion criteria include elevated fasting plasma glucose (= 6.0 mmol/L), elevated 2 hour plasma glucose (= 11.1 mmol/L) after a standard glucose load, A1C > 6.0%, and contraindications to metformin or exercise and inability to engage in group activities.

### General Procedure

#### Screening Visit

**Figure 1:**
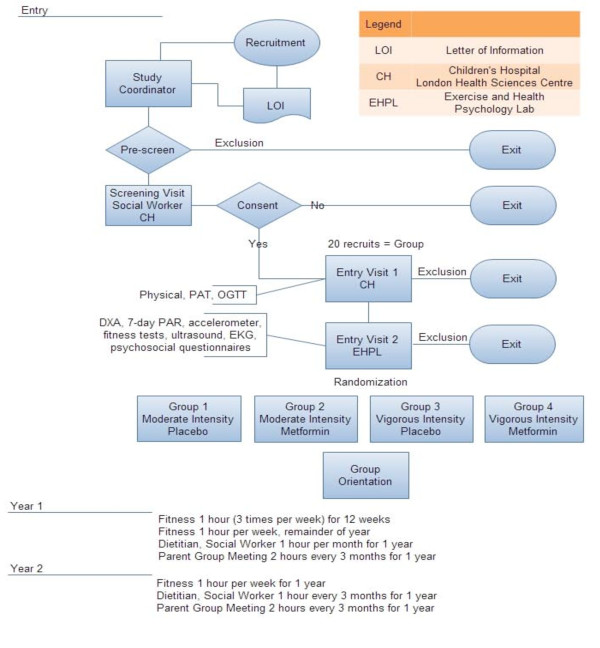
**Flow Diagram of REACH protocol**.

During the initial screening visit, the parent and child meet with a social worker who explains the details of the study and what is expected from the family. The social worker assesses both child and parent motivation, and establishes parental commitment to be their child's 'coach' for the entire program. Participants then complete informed consent and sign up for Entry Visits 1 and 2 (see Figure 1 for a diagram of the procedure).

#### Entry Visit 1 - Medical History and Physical Examination

The first entry visit occurs at the Children's Hospital between 1-6 weeks before the 12-week intensive exercise program begins. Participants are fasting and have endothelial function measured by PAT (peripheral arterial tonometry) and an oral glucose tolerance test (GTT) with measurement of thyroid stimulation hormone (TSH), transaminases (AST and ALT), blood urea nitrogen (BUN) and creatinine, HDL and LDL cholesterol, triglycerides, adiponectin and leptin in the fasting blood sample. A physical examination is completed by a pediatrician and includes pubertal staging and documentation of presence of acanthosis nigricans, hirsutism and acne. During this visit, potential risk factors for development of T2D are assessed; including family history of T2D, gestational diabetes, birth weight, and ethnicity. Objective measures of height and weight are taken in order to determine BMI.

#### Entry Visit 2 - Body Composition, Fitness and Psychosocial Assessments

This visit takes place at the Exercise and Health Psychology Laboratory (EHPL-http://www.ehpl.uwo.ca) at the University of Western Ontario (UWO) and occurs between 1-6 weeks before the 12-week intensive exercise program begins. During the second entry visit, participants complete a number of assessments including: psychosocial questionnaires (described in Measures), ultrasound, electrocardiography, body composition, upper and lower body strength and aerobic fitness. Participants are then given an Actical™ accelerometer to wear for 1 week to obtain an objective measure of physical activity at baseline. After wearing the Actical™ for one week, participants return it to the EHPL and complete the 7-day physical activity recall [39] for the previous week.

#### Progress Assessments at 2-, 6-, and 13 weeks, 6-months, 1- and 2-years

See Table 1 for list of assessments completed at each time point.

**Table 1:** Summary of Measure Assessments

	Assessment Period							
Measure	E1	E2	2 weeks	6 weeks	13-weeks	6 month	1 year	2 years
Height, weight	Monthly						Every 3 months	
DXA		X			X	X	X	X
Waist Circumference	Monthly						Every 3 months	
Blood Pressure				X	X	X	X	X
Aerobic fitness		X			X	X	X	X
Strength fitness		X			X	X	X	X
Actical™		X			X	X	X	X
7-day PAR		X			X	X	X	X
Ultrasound		X			X	X	X	X
EKG		X			X	X	X	X
PAT	X					X	X	X
Blood work	X					X	X	X
3-day food records				X	X	X	X	X
Quality of Life		X			X	X	X	X
Social Support		X			X	X	X	X
Outcome Expectations		X		X	X	X	X	X
Satisfaction		X		X	X	X	X	X
Enjoyment		X		X	X	X	X	X
Self-efficacy			X	X	X	X	X	X
Group Cohesion			X		X			
Collaboration			X		X			
Intentions			X	X	X	X	X	X

### Sample size and statistical analyses

The sample size will be 30 patients per medication group (metformin or placebo), calculated to have an 80% power of detecting a difference of 1.5 in BMI, assuming an alpha of 0.05, and a standard deviation of 2^70^. Allowing for a 20% drop out rate, 72 subjects will be recruited. The means and standard deviations of BMI scores and the secondary outcomes will be compared between the two treatment groups using two-tailed tests. Results obtained at two separate time points within a group will be compared by paired Student T-test, or for non-parametric data using the Wilcoxon test. Comparison of results between different patient groups will be performed using non-paired Student T-test, or a Mann-Whitney U test for non-parametric data. Comparisons between multiple time points will utilize ANOVA repeated measures tests.

### REACH Intervention

The REACH program is a multidisciplinary, family-based childhood obesity treatment program. The behavioural interventions in REACH are based on Bandura's [9] social cognitive theory (SCT), and Epstein's [11] model of multidisciplinary, family-based interventions. The REACH multidisciplinary health care team is comprised of a behaviour change interventionist, exercise trainers, a dietitian, a social worker, a pediatrician and a study coordinator. Both parents and children are involved in the program to encourage healthy, sustainable changes in the home environment. The REACH program takes place at both the Children's Hospital, London Health Sciences Center and the Exercise and Health Psychology Lab (EHPL) at The University of Western Ontario. The program delivered by each health care provider will be described in detail below.

### 12-Week Intensive Program

#### Exercise Intervention

For the first 12 weeks of REACH, all participants exercise at the EHPL for 1 hour, 3×/week for weeks 1-6; 1 hour, 2×/week for weeks 7-9; and 1 hour 1×/week for weeks 10-12. The exercise sessions are progressively less frequent to facilitate participants incorporating *independent *physical activity into their own daily schedule. As a reward for their hard work, after the last session of the intensive 12 weeks, participants have an exercise session at the local community center activity room, in which they are able to play active video games and have group relay races with an interactive wall. Participants are randomized into either a moderate or a vigorous intensity exercise program. Both moderate and vigorous intensity exercise sessions include a warm-up, aerobic exercises, resistance training and cool down (see Table 2 for break down of time spent in group sessions, cardio exercise and resistance training).

**Table 2:** Time spent in Behaviour Change, Cardio Exercise and Resistance Training

DAY	GROUP 1	GROUP 2
**1**	4:30-4:50 pm - behaviour change session 4:50-5:10 pm - cardio exercise 5:10-5:30 pm - resistance training	5-5:20 pm - behaviour change session 5:20-5:40 pm - cardio exercise 5:40-6 pm - resistance training
**2**	4:30-5 pm - cardio exercise 5-5:30 pm - resistance training	4:30-5 pm - resistance training 5-5:30 pm - cardio exercise
**3**	4:30-5 pm - resistance training 5-5:30 pm - cardio exercise	4:30-5 pm - cardio exercise 5-5:30 pm - resistance training

##### Cardiovascular exercise sessions

During the cardiovascular exercise sessions, participants exercise on treadmills, steppers, rowers and bikes. All participants wear heart rate monitors (Polar Electro Oy, Kempele, Finland) and are given instructions to keep their heart rate at a specific intensity (within a range of 10 bpm; see Table 3 for intensities). Participants are also asked to report their rate of perceived exertion (RPE) as a subjective measure of physical exertion [40]. Participants in the moderate intensity group aim to be at 4-6 RPE, and the vigorous group at 7-9 RPE. Heart rate intensities are adjusted to ensure the participants are in the correct RPE range for their respective group assignment (see Table 3 for details). The heart rate data are downloaded and analyzed to serve three purposes: 1) As a manipulation check to ensure participants are exercising at their prescribed intensity, 2) as motivation, and 3) to track progress. To enhance motivation, for each session participants achieve their target exercise intensity, they are rewarded by being granted permission to use their own personal music device (i.e., iPod, MP3 player) to exercise with in the next session.

**Table 3:** Summary of 12-week Exercise Program

Training	Cardio	Resistance
**Exercises**	Treadmill	Leg press
	Stationary bike	Lat pull
	Stepper	Leg abduction and adduction
	Rower	Tricep/bicep curls
		Chest press
		Lunges
		Sit-ups
		Back extensions
		Side bends
		Push-ups
		Should raises
		Skipping
**Intensity**	*Moderate:*	*Moderate:*
	40-59% HRR*	1-2 sets × 15 repetitions
	*Vigorous:*	*Vigorous:*
	60-79% HRR*	1-2 sets × 10 repetitions

*HRR calculated by ((HRmax-HRresting)*intensity) + resting HR

##### Resistance training sessions

During the resistance training sessions participants exercise on pneumatic resistance machines (HUR, Finland) and complete floor and free weight exercises (see Table 3 for details).

##### Group-mediated cognitive behavioural sessions

For the first 12 weeks of the intervention, participants engage in weekly group behaviour change sessions. These sessions are delivered in a group setting, and are based on the group-mediated cognitive-behavioural (GMCB) intervention model [17]. These sessions occupy the first 20 minutes of each Monday session (see Table 4 for session content). The purpose of these sessions is to help participants develop the self-regulatory skills necessary to become and remain physically active after the study has ended. Self-regulatory skills sessions involve teaching participants how to self-monitor, set goals, plan regular physical activity and overcome barriers to physical activity. For example, participants monitor their daily physical activity behaviour and the group brainstorms ways to overcome barriers to physical activity that are effective for them.

**Table 4:** Summary of Behaviour Change Session Content

WEEK	SESSION CONTENT	EXPLANATION
**1**	Developing group cohesion	Come up with team name and cheer, discuss benefits of being part of a team.
	Self-monitoring	Learn how to use a pedometer and physical activity log. Monitor activity over next week.

**2**	Self-monitoring	Discuss activity over last week. Continue to monitor.
	Continue to foster group cohesion	Set up the buddy system (check in with other group member during week to discuss physical activity).

**3**	Goal setting Group cohesions	Aware of current activity, set individual and group goal based on FITT principal.

**4**	Overcoming barriers to physical activity	Discuss achieving goal. Anything that stopped them? Brainstorm how to overcome.

**5**	Rewards Living balanced lifestyle	How to use rewards to help motivate Look at active/inactive activities in a day

**6**	Review	Jeopardy-style review of what have learned so far

**7**	Planning	Learned to set daily plan for physical activity

**8**	Exercise at home Set new PA goal	Develop exercise plan can do at home

**9**	Overcoming lapses	Discuss how to get back on track

**10**	Keys to success	Discuss cues can use to remind about and stay motivated for daily physical activity Discuss and practice thought stopping and positive self-talk

**11**	Review	Review all behaviour change skills learned over past 12 weeks. Discuss how every skill can help each person.
**12**	Review	

### 2-Year Lifestyle Program

#### Weekly Exercise Sessions

After completing the intensive 12-week exercise program at the EHPL, participants enter the long-term weekly exercise program. This program involves weekly exercise sessions with the entire group (i.e., moderate and vigorous groups combined) at a local community center. Participants engage in a variety of different activities including playing games and sports, going swimming and attending group exercise classes together. The purpose of these sessions is to keep the participants accountable and to help them maintain their active lifestyle. Participants are given a 2-year membership to a community center to encourage daily physical activity.

#### Family Sessions

Over the course of the 2-year study there are eight family sessions. The purpose of these sessions is to teach children and parents healthy behaviours and maintain contact in a fun environment (see Table 5 for summary of family sessions).

**Table 5:** Summary of Group Family Sessions

SESSION	ACTIVITY	EXPECTED OUTCOMES
**1**	Orientation Night	Chance for participants to meet REACH health care team and each other.

**2**	Grocery Store Tour	Dietitian runs this tour, help families learn to read labels, shop for healthy food within a budget

**3**	Jeopardy Night	Review what families have learned in a fun and (slightly!) competitive environment.

**4**	Cooking Class	Dietitian runs this cooking class. Chance for participants to learn how to cook easy, healthy meals.

**5**	Outdoor Activity	Have fun being active together outside

**6**	Community Center Activity Night	Have fun being active together as a family and with other participants

**7**	Inspirational Speaker	Motivate participants to continue to make and maintain healthy lifestyle changes

**8**	Potluck Dinner	Celebration after completing 2 years of the study. Chance for participants to bring some of their favorite healthy meals to share with each other.

#### Clinical Visits

Participants are required to attend clinical visits with the dietitian and social worker at the Children's Hospital, London Health Sciences Centre, beginning at the end of month one of the intervention. Participants are seen monthly for the first year and every 3 months during the second year and spend approximately 30 minutes each with the dietitian and the social worker, for a total visit time of one hour. Visits are held during evening clinic hours so as to not compromise the school and work schedules of the participants and families involved. Clinical visits are focused on providing family-centered care, thus meetings involve at least one parent or caregiver and the participant. Caregivers are experts in relation to their child and family, are valuable resources when determining if certain goals or lifestyle changes are realistic, and can be motivating agents of change for the family. Similarly, because the family is the child's primary social learning environment, collaborating with the child and family can encourage family-wide changes, increase time spent together as a family, and empower each other to make and maintain healthy changes. Details of the sessions with the dietitian and the social worker will be discussed.

#### Nutrition Sessions with the Dietitian

Participants and families are encouraged to follow "Eating Well with Canada's Food Guide" [41], as it promotes healthy eating based on nutrient standards and the prevention of chronic diseases such as T2D, CVD and certain types of cancer [41]. Measurable and achievable nutrition goals are set collaboratively by the dietitian, participant and family to increase fruit and vegetable servings and whole grain products, encourage lower fat options such as 1% or skim milk and alternatives, and limit high calorie, low nutrient dense foods such as sodas, fruit drinks, candy and highly processed foods. These recommendations are congruent with "Eating Well with Canada's Food Guide", and when implemented in combination with regular physical activity, have been shown to be effective in achieving and maintaining a healthy weight while decreasing the likelihood of chronic diseases [[42]; see Table 6 and 7 for summary of nutrition sessions].

**Table 6:** Summary of Sessions with Dietitian

**Foundational Nutrition Sessions (2)**	
**Month 1**	Eating Well with Canada's Food Guide
	Review of serving sizes and food groups
	Strategies to meet recommended amounts
	Proper meal spacing

**Month 2**	Balance and variety for meals
	3-4 food groups per meal
	Healthy snacking (combining 2 food groups)

**Table 7:** Summary of Sessions with Dietitian

**Elective Nutrition Sessions (14)**	
Taught at the discretion of the dietitian and therefore may include but not limited to curriculum below	
Timing and detail may be modified to meet individual needs	
**Month 3-24**	Hunger, satiety cues and picky eaters
	Nutrition Facts Tables
	Healthy eating versus fad diets
	Menu planning, shopping and budgeting
	Sports nutrition
	Heart healthy eating (cholesterol, sodium and healthy fats)
	Hydration and fluids (water and milk versus energy and sports drinks)
	Vitamins, minerals and supplements
	Sugars and sugar substitutes
	Strategies for eating out and holiday foods
	Soluble versus insoluble fibre (strategies to meet fibre recommendations)
	Recipe Makeover (making recipes healthier, nutrition websites)
	Glycemic Index
	Home made versus processed foods (creative, healthy ideas for back to school lunches and snacks)

#### Psychosocial Sessions with the Social Worker

The role of the social worker is to provide age-appropriate education, counseling and intervention to improve health status and enhance psychosocial functioning of the participants and families involved in the study. The social worker strives to increase each participant's quality of life, self-esteem and sense of value, strengthen his/her social relationships, and address social barriers to physical activity and healthy eating. In consideration of best practices, sessions are flexibly structured and focus around 5 core themes: assessment, goal-setting, problem-solving, coaching, and support (see Table 8 and 9 for summary of psychosocial sessions).

**Table 8:** Summary of Sessions with Social Worker

**Foundational Psychosocial Sessions (2)**	
**Month 1**	Assess participant and family in relation to commitment, motivation, support available
	Identify participant's areas of need, interest, talent and strength
	Clarify participant's psychosocial goals
	Introduce concept of healthy goal-setting, emphasizing that goals should be realistic and achievable

**Month 2**	Social barriers to changing lifestyle
	Family response to barriers (coping strategies, problem-solving)
	Celebrating success with appropriate reward systems
	Increase understanding of behaviour modification techniques (monitor thoughts and feelings to increase self-control)
	Ongoing provision of support and encouragement (positive reinforcement, encourage family involvement)

**Table 9:** Summary of Sessions with Social Worker

**Elective Psychosocial Sessions**	
Taught at the discretion of the social worker and therefore may include but not limited to curriculum below Timing and detail may be modified to meet individual needs	
**Month 3-24**	Self-esteem
	Body image
	Media and peer pressure
	Teasing/bullying/social exclusion/stigmatization
	Coping strategies and problem-solving
	Increasing family time/activities/involvement
	Increasing and enhancing family communication
	Sustainable change
	Increasing ownership and responsibility in study
	Scheduling and time management
	Financial constraints (cost-effective strategies for healthy living)

##### Assessment

Prior to consenting, the social worker meets with the participant and family to assess their readiness for and commitment to change. Throughout the subsequent sessions, issues related to commitment, motivation, challenges, barriers to success, and on-going or changing need areas are discussed. Intervention is flexible as areas of need are addressed when they surface.

##### Goal-setting

Setting and working toward goals helps participants take ownership for their involvement and empowers them to make healthier choices at home. Some goals may be collective family goals and other goals are individualized for the participant. The social worker assists participants and families to ensure that the goals are realistic and achievable. At each session, the participant and family discuss progress and challenges in working toward the goals agreed upon at the last session. New goals are created in each session and the participant and family identify the steps necessary to work toward achieving them. Goals are written on a handout entitled "REACH Action Plan", which participants receive at the end of each session. This helps to formalize the goals and enables participants to remember what was discussed and what goals they have committed to working toward.

##### Problem-solving

Participants often experience challenges, concerns and barriers throughout their involvement in this study. Specifically, concerns often surface in relation to decreased levels of motivation, unpredictable family involvement, scheduling difficulties and diverse family perspectives, and these can compromise the participant's engagement in the study. The social worker helps to normalize these challenges while addressing this resistance in a solution-focused manner.

##### Coaching

The social worker acts as a 'coach' in relation to lifestyle or parenting strategies. As some research suggests that the self-esteem of children who are obese is associated with social experiences, the manner in which family members respond to weight issues, and the family's development of effective coping skills and strategies [43] are important points of focus. Recommended practices in obesity programming include being family-centered and focusing on parental skill-building [44]. In practice, 'coaching' may include discussing positive ways to celebrate progress or success, identifying appropriate reward systems, and assisting caregivers how to use praise and encouragement. Ultimately, the goal is to increase family involvement and constructive communication, which will have a positive impact on the participant's self-esteem and quality of life.

##### Support

The social worker provides different levels of support based on self-identified needs of the participant and family. When participants are afforded the opportunity to identify and communicate what they need to succeed, they are more likely to take responsibility and ownership for their needs. As such, the level of support, encouragement and assistance is dependent on the communicated wishes of the child and family. Through role-modeling positive reinforcement and support for the family, it is hoped that the family will adopt such practices and provide an accepting environment wherein the risk of stigmatization for the participant is decreased [45].

#### Metformin Therapy

Until recently, metformin was available in a relatively short-acting formulation at a dose of 500 mg or 850 mg necessitating multiple daily dosing. Metformin is now available in an extended release formulation facilitating once daily dosing that is likely to be associated with improved compliance. All participants start taking the pills on day 1 of the study intervention. Participants begin therapy at 500 mg/day of metformin or placebo and increase by 500 mg/day every 7 days to a maximum tolerated dose of 2000 mg/day, taken before the evening meal as a single daily dose. The metformin preparation is Glumetza (metformin HCL), available in 500 mg extended release pills. The study co-ordinator monitors participants' drug tolerance and compliance weekly for the first 5 weeks, and then every 28 days for the remaining 21 months. Monthly pill counts are done throughout the study.

### Measures

#### Primary Outcome

##### Body mass index (BMI)

BMI is calculated based on objective measurements of height and weight. Using the age- and gender- specific BMI references developed by the Center for Disease Control and Prevention [46], BMI is expressed as a z-score (as a standard deviation score). In children, BMI z-scores are a more reliable comparison measurement among participants than absolute BMI [46]. Change in BMI z-score is the primary outcome measure.

#### Secondary Outcomes

##### Body composition assessments

*Fat mass, fat free mass, and percent body fat.* A dual-energy x-ray absorptiometry (DXA) scan (General Electric-Lunar iDXA, Ames Medical) is used to obtain whole body fat, lean muscle mass and fat-free mass assessments.

*Waist circumference.* Waist circumference is measured in the standing position at the level of the umbilicus to the nearest 0.1 cm using a constant tension measuring tape. The reading is taken without the tape compressing the skin.

##### Metabolic Assessments

*Glycemic status.* Glycemic status is assessed by insulin resistance using HOMA (Homeostasis Model Assessment) and 2-hour plasma glucose level after a standard glucose load. HOMA is calculated as fasting plasma insulin (mU/L) × fasting serum blood glucose (mmol/L)/22.5). Impaired glucose tolerance (IGT) is defined as a 2-hour plasma glucose level of >7.8 mmol/L and <11.1 mmol/L, after a standard glucose load.

*Serum lipids.* Fasting levels of HDL cholesterol, LDL cholesterol and triglycerides are obtained.

*Plasma adipocytokines.* Leptin and total adiponectin levels in serumare are measured by specific, commercial ELISAs (Quantikine, R&D Systems, Minneapolis, MN).

##### Vascular Assessments

*Blood pressure.* The participant rests their arm elevated at heart level, supported by a pillow, for 5 minutes prior to taking their blood pressure.

*Endothelial function.* Endothelial function is assessed using peripheral arterial tonometry (PAT, Itamar Medical Ltd). PAT is a non-invasive technology, which uses pneumatic probes designed to measure pulsatile volume changes at the fingertips. These probes cap the finger over the distal phalanx and apply a uniform pressure field around the digit, which allows for measurement of the pulsatile oscillations of the digital vascular bed microcirculation. PAT testing takes 15 minutes and is non-invasive and radiation free. Continuous recordings (PAT data) are stored and analyzed by computer using an automated algorithm to calculate the reactive hyperemia PAT index (RH-PAT) for each participant. Lower RH-PAT index scores are reflective of greater endothelial dysfunction and risk for atherosclerosis. Normative data for RH-PAT is available from previous studies [47,48].

*Vascular properties.* Major conduit vessels is examined because these provide the major buffering capacity for systolic pressure and they provide enhanced spatial resolution for analysis. Using echo Doppler imaging (GE Vivid 7; 10 MHz), the wall thickness (anterior and posterior wall), intima-media thickness, and cross-sectional diameter of each of the carotid, femoral and brachial arteries is measured at systole and again at end diastole. Measures of arterial blood flow (Doppler ultrasound; 4.7 MHz) are collected to determine regional blood flow and thereby calculate downstream vascular resistance (the quotient of mean arterial pressure and blood flow). Combining the vascular diameter data with the measured systolic and diastolic pressure, the distensibility of the respective vessels is quantified using the following equation: D = 2 × (systolic diameter - diastolic diameter)/(diastolic diameter/[systolic BP - diastolic BP]), where D is distensibility and BP is the blood pressure.

*Heart Variability. * The electrocardiogram is collected for approximately 10 minutes while the participant rests quietly in the supine position. Using the R-R intervals, heart rate variability is quantified with emphasis on the high frequency power (0.015 - 0.25 Hz), which is interpreted to reflect neural control of the heart, particularly the parasympathetic nervous system [49-51].

##### Program Adherence

*Attendance.* Attendance for each subprogram (i.e., exercise, social work, nutrition) is calculated by dividing the number of sessions the child (and parent if applicable) attended, by the number of sessions scheduled. Number of missed appointments is documented.

*Medication.* Pill counts are taken monthly to monitor compliance for taking the medication (metformin or placebo). Compliance is calculated as a percentage of pills that should have been taken versus number returned every 28 days.

##### Physical Performance Assessments

*Aerobic fitness.* Aerobic fitness is evaluated using the modified Balke incremental exercise protocol on a Woodway PPS treadmill [52]. Expired gases are analyzed using a metabolic cart (Cosmed Quark B^2 ^cardiopulmonary testing, Image Monitoring). Peak oxygen consumption (Peak VO_2_) expressed in ml/kg/min is determined by taking the highest values during a 15 second period. The test is terminated when participants indicate they are unable to continue the protocol, or when their vital signs warranted.

*Strength.* Isometric strength is assessed during the leg press and chest press using a strain gauge manometer connected to the respective HUR machines. Three maximum voluntary contractions (MVC) are performed of 3-5 second duration for the right leg and arm with 1-minute rest between each contraction. The maximal value is stored as the peak power. Participants are provided visual feedback of the force, and verbally encouraged [53]. Strength values of the peak MVC are used for analyses.

##### Exercise Intensity Assessments

*Heart rate.* In order to ensure participants are exercising at the correct intensity (moderate or vigorous), all participants wear the Polar Vantage XL (Polar Electro Oy, Kempele, Finland) heart rate monitor during the aerobic exercise sessions in the intensive 12-week program. Heart rate data is downloaded every session and average heart rate for each participant is recorded.

*Rating of perceived exertion.* The OMNI Rating of perceived exertion (RPE) scale [54] is used during the aerobic exercise portion of the study to determine the participant's subjective ratings of how hard they are working. The scale ranges from 0 (*not hard at all*) to 10 (*very very hard*). Participants in the moderate intensity group are asked to adjust their intensity so that they are between 4-6 on this scale, and the vigorous group are between 7-9. This scale is a reliable indicator of physical effort in children and has sound psychometric properties [40,54].

##### Physical Activity Assessments

*Self-reported physical activity.* Participants complete the interview version of the 7-day Physical Activity Recall (PAR) Questionnaire [39] to assess how much PA (frequency, intensity and duration) they engaged in over the past week. The interviewer asks participants about the activities they engaged in each day during the morning, afternoon and evening. The participant is also asked to report how long they engaged in each activity, and what intensity it was (i.e., light, moderate, hard or very hard). This questionnaire has been shown to have satisfactory test-retest reliability and has been validated by comparing with heart rate monitor records in adolescents [55].

*Objective physical activity.* An objective measure of PA is obtained using the Actical™ activity monitor (Respironics, Mini Mitter). Participants wear this device for one week at baseline and at all follow-up assessment periods. Activity is counted when participants wear the actical for 10 hours per day for 5 days [56]. The validity and reliability of this measure in youth has been confirmed [57].

##### Psychosocial Function Assessments

All measures that have not been previously validated with adolescents were pilot tested with youth the same age as the REACH participants. Measures modified based on previously validated measures were adapted to use appropriate terminology for youth.

*Quality of life.* Pediatric Quality of Life Inventory [Peds-QL; [58]] is used to assess children's quality of life. This 23-item self-report questionnaire includes four subscales to assess the child's physical, social, emotional and academic functioning and includes items such as "In the past one month, how much of a problem has it been for you to do sports activities or exercise?" Quality of life is quantified as the participant's mean score across all four subscales. Each item is rated on a 5-point scale from 0 (*Never*) to 4 (*Almost Always*). Scores are linearly transformed to a 0-100 scale as follows: 0 = 100, 1 = 75, 2 = 50, 3 = 25 and 4 = 0, such that higher scores on this scale indicate higher functioning. Varni and colleagues [59] reported that across a large sample size, this scale exceeded the minimum internal reliability criterion of 0.70, and had satisfactory validity because the Peds-QL was able to distinguish between children with chronic conditions and healthy children.

*Social support.* Social support is assessed using a 7-item scale developed for this study. This scale examines the degree to which children feel their parents support them to engage in physical activity. This scale includes items such as "During a typical week, how often did your parent(s) or family members encourage you to do physical activities or play sports?" Each item is rated on a 5-point scale from 0 (*Never*) to 4 (*Every day*). Higher scores on this scale indicate a higher level of social support.

*Outcome expectations.* Participants' beliefs that engaging in regular physical activity will lead to specific valued outcomes is assessed using a 36-item outcome expectations questionnaire [modified for youth from Rogers & Brawley; [60]]. Outcomes are divided into three categories: social, physical and psychological. Participants are asked to rate the likelihood and value of the outcome occurring as a result of participating in physical activity over the next four weeks. This scale is assessed on a 9-point Likert response scale, from 1 (*very unlikely or low value*) to 9 (*very likely or highly value*). Sample items include: "If I participated in physical activity over the next 4 weeks, the likelihood of it being fun is..." and " It is important to me that physical activity is fun". Cronbach's alpha measure of internal validity for this scale has been found to be acceptable [alpha = .88; [60,61]].

*Satisfaction.* Participants' satisfaction is assessed using measures modified for children based on Jeffery and colleagues [62]. There are three categories of satisfaction assessed in this study: satisfaction with current state, satisfaction with progress, and satisfaction with changes resulting from participating in the REACH program. Participants are asked to rate how satisfied they are with the outcome described and respond on a 9-point Likert response scale, from -4 (*very unsatisfied*) to 4 (*very satisfied*). A sample item is "How satisfied are you with how intensely you can engage in physical activity?" Cronbach's alpha measure of internal validity for this scale has been found to be acceptable [alphas > .76; [61,62]].

*Enjoyment.* Participants' enjoyment of PA is assessed using the *Physical Activity Enjoyment Scale *[PACES; [63]]. This scale has 18 Likert-type items and is used to assess enjoyment of the PA the participant has engaged in over the last week. A sample item is "Physical activity over the past week for me has been:" The participant responds on a 7-point Likert response scale, from 1 (*I hate it*) to 7 (*I enjoy it*). Exercise enjoyment is computed by determining the scale average score. Motl [64] reported that PACES had acceptable levels of internal consistency [alpha = .92; [61]].

*Self-efficacy.* In order to accurately assess self-efficacy, several domains need to be evaluated [65]. Furthermore, self-efficacy should only be assessed after the participant has had a chance to try the behaviour so that answers are not over or under-estimated [65]. In this study, task self-efficacy and selected targeted aspects of self-regulatory efficacy (goal setting, planning and barriers) are assessed using measures based on those developed by Cramp and Brawley [21] for their GMCB exercise intervention study. All four scales are scored on a 100 percent confidence scale, from 0 percent (*Absolutely Not Confident*) to 100 percent (*Absolutely Confident*) scale, in 10 percent increments. Self-efficacy for each scale is computed by determining the scale average score.

*Task self-efficacy.* The purpose of this 9-item questionnaire is to assess participant's confidence in their ability to engage in increasing intensities and durations of physical activity. A sample item is: "How confident are you that you can complete 10 minutes of physical activity at a light intensity three times next week?" Cronbach's alpha measure of internal validity for this scale has been found to be acceptable [alpha = .83; [61,66]].

*Goal setting self-efficacy.* The purpose of this 5-item questionnaire is to assess participant's confidence in their ability to set goals to be physically active. A sample item is: "How confident are you that you can set realistic goals for increasing and maintaining your physical activity in the next month?" Cronbach's alpha measure of internal validity for this scale has been found to be acceptable [alpha = .97; [21,61]].

*Planning self-efficacy.* The purpose of this 7-item questionnaire is to assess participant's confidence in their ability to plan and schedule regular physical activity. A sample item is: "The amount that I am confident that I could arrange my schedule to be physically active on my own is..." Cronbach's alpha measure of internal validity for this scale has been found to be acceptable [alpha = .87; [21,61]].

*Barriers self-efficacy.* The purpose of this 16-item questionnaire is to assess participant's confidence in their ability to overcome barriers to engaging in physical activity. A sample item is: "How confident are you that you could engage in physical activity even if you have a lot of school work to do?" Cronbach's alpha measure of internal validity for this scale has been found to be acceptable [alpha = .82; [21,61]].

*Behavioural intentions.* Participants' intentions to engage in the organized exercise classes and independent PA is assessed using a 4-item measure of participant self-prediction [67]. Participants indicate the number of times they intend to attend the REACH exercise class over the course of the next four weeks. Next participants rate how strongly they feel they will attend this number of classes on a 1 (*definitely will not*) to 9 (*definitely will*) scale as suggested by Fishbein and Stasson [67]. Participants are also asked to indicate how many days they will engage in PA on their own over the next week, in addition to the exercise program. Once again, participants then indicate how strongly they feel they would engage in this number of independent PA sessions on a 1 (*definitely will not*) to 9 (*definitely will*) scale.

*Group cohesion.* One of the central tenants of GMCB interventions is that the group setting facilitates participants' learning of self-regulatory skills. This questionnaire was modified for youth based on Cramp & Brawley [21] and is administered in order to assess if the group environment has obtained a strong level of group cohesion. This measure assesses groupness and task related cohesion. A sample item is "The group members help keep everyone motivated to continue being physically active". Participants respond to questions on a Likert 5-point scale from 1 (*strongly disagree*) to 5 (*strongly agree*).

*Collaboration.* In GMCB interventions it is also important to assess the collaboration between the group and the interventionist. This questionnaire is administered to assess collaboration between the group and both the group session leader and the exercise leader and is modified for youth based on Cramp & Brawley [21]. A sample item for the group session leader collaboration is "I feel our discussion leader wants to know about our opinions and values our opinions about fitting the skills we learned into our daily life", and for exercise session leader collaboration is "Our physical activity leader cares about my healthy and about my opinions for developing my own physical activity program". Responses for each item are on a Likert 5-point scale from 1 (*strongly disagree*) to 5 (*strongly agree*).

##### Nutrition Assessments

The following nutrition assessment tools document food intake over a period of time and when analyzed, provide estimates of nutrients and number of food guide servings. Based on these data, age appropriate and family centered nutrition recommendations are discussed.

*Diet.* During the initial meeting the dietitian conducts an overall diet assessment. This assessment involves a series of open-ended questions. The dietitian inquires about any previous diet counseling, recent changes in nutrition habits, allergies, medications and/or supplements, preparation and location of meals, social determinants of health (i.e., financial barriers), as well as behavioural and emotional attitudes toward food. The purpose of the nutrition assessment is to provide an opportunity for the dietitian and family to become acquainted and to gather information that allows the dietitian to provide individualized family-centered nutrition care plans.

*3-day Food Record.* The participants are expected to complete a 3-day food record prior to each visit with the dietitian. The 3-day food record is a one-page chart on which participants write down all meals, snacks and beverages including estimated amounts (i.e., 1 1/2 cups of oatmeal). This tool is seen as a gold standard when measuring food intake in adults and a preferred choice when measuring food intake in children, although it requires a higher level of literacy, motivation and cognitive ability of the participant [68]. Participants and family members are prompted on incomplete food records and, should the participant fail to present a 3-day food record, the dietitian administers a 24-hour recall, as children are thought to be able to accurately respond [69].

## Discussion

In summary, this article has provided a rationale for the REACH program, including a detailed overview of the multidimensional intervention program for obese children and their families. Furthermore, detailed description of all primary and secondary outcome measures are described. All of the information needed to replicate a similar intervention program has been documented. To date, 31 obese children and their family members have met the eligibility criteria and are completing different stages of the REACH program. Preliminary results of the 12-week intervention program (with the remaining 42 obese children and their families) are expected in late 2010, with complete 2-year intervention data available in late 2012. Findings from the REACH program will be disseminated at national and international scientific meetings and to relevant community and government organizations. Findings will also be published in academic journals. In the meantime, we hope that the issues discussed provide guidance to those undertaking similar trials with obese children.

## Competing interests

The authors declare that they have no competing interests.

## Authors' contributions

Principal responsibility for study design and conduct was assumed by CLC. AJW, HP, JV, LL, MEJ, ACG, MW, TR, and KS contributed to the study design. AJW drafted the manuscript. All authors read and commented on drafts and approved the final manuscript.

## Pre-publication history

The pre-publication history for this paper can be accessed here:

http://www.biomedcentral.com/1471-2458/9/434/prepub
